# Differential changes in self-reported aspects of interoceptive awareness through 3 months of contemplative training

**DOI:** 10.3389/fpsyg.2014.01504

**Published:** 2015-01-06

**Authors:** Boris Bornemann, Beate M. Herbert, Wolf E. Mehling, Tania Singer

**Affiliations:** ^1^Department of Social Neuroscience, Max Planck Institute for Human Cognitive and Brain SciencesLeipzig, Germany; ^2^Clinical Psychology and Psychotherapy, University of TuebingenTuebingen, Germany; ^3^Health Psychology, University of UlmUlm, Germany; ^4^Department of Family and Community Medicine, Osher Center for Integrative Medicine, University of California San FranciscoSan Francisco, CA, USA

**Keywords:** interoceptive awareness, interoception, body awareness, contemplative training, meditation, questionnaire, change, mindfulness

## Abstract

Interoceptive body awareness (IA) is crucial for psychological well-being and plays an important role in many contemplative traditions. However, until recently, standardized self-report measures of IA were scarce, not comprehensive, and the effects of interoceptive training on such measures were largely unknown. The Multidimensional Assessment of Interoceptive Awareness (MAIA) questionnaire measures IA with eight different scales. In the current study, we investigated whether and how these different aspects of IA are influenced by a 3-months contemplative intervention in the context of the ReSource project, in which 148 subjects engaged in daily practices of “Body Scan” and “Breath Meditation.” We developed a German version of the MAIA and tested it in a large and diverse sample (*n* = 1,076). Internal consistencies were similar to the English version (0.56–0.89), retest reliability was high (*r*s: 0.66–0.79), and the MAIA showed good convergent and discriminant validity. Importantly, interoceptive training improved five out of eight aspects of IA, compared to a retest control group. Participants with low IA scores at baseline showed the biggest changes. Whereas practice duration only weakly predicted individual differences in change, self-reported liking of the practices and degree of integration into daily life predicted changes on most scales. Interestingly, the magnitude of observed changes varied across scales. The strongest changes were observed for the regulatory aspects of IA, that is, how the body is used for self-regulation in daily life. No significant changes were observed for the Noticing aspect (becoming aware of bodily changes), which is the aspect that is predominantly assessed in other IA measures. This differential pattern underscores the importance to assess IA multi-dimensionally, particularly when interested in enhancement of IA through contemplative practice or other mind–body interventions.

## INTRODUCTION

Interoceptive awareness (IA) comprises the awareness of signals from the inside of the body, such as the perception of heart beats, the breath, or movements of the viscera, and higher-order top–down processes including biases, beliefs, attitudes, and emotions regarding those perceptions (Cameron, 2001; Craig, 2002; Mehling et al., 2009). Interoception has been shown to be critical for the sense of self and the creation of a subjective perspective from which the world is experienced (Varela et al., 1991; Craig, 2002, 2009; Critchley et al., 2004; Berlucchi and Aglioti, 2010; Park and Tallon-Baudry, 2014). It is also important for awareness and regulation of emotions (Dunn et al., 2007; Silani et al., 2008; Herbert et al., 2011; Füstös et al., 2013; Koch and Pollatos, 2014) as well as empathy (Singer et al., 2009; Bird et al., 2010; Lamm and Singer, 2010; Terasawa et al., 2014). Furthermore, IA is critical for decision making (Sanfey et al., 2003; Dunn et al., 2010; Sütterlin et al., 2013) and self-control of behavior in various situations with impact on health and disease (Herbert et al., 2007, 2012a, 2013; Herbert and Pollatos, 2014).

Contemplative traditions have also widely recognized the importance of IA (Selby, 1992; Goldstein and Kornfield, 1995; Vaughan, 2002; Hölzel et al., 2011; Kerr et al., 2013) and devised mental training practices, such as bodily focused meditations, to train awareness of body sensations (Hart, 1987; Kabat-Zinn, 1990). Despite the importance that malleability of IA could have due to its potential association with beneficial psychological and physical outcomes, few studies have investigated whether and how different aspects of IA are influenced through mental training.

Interoceptive awareness can be assessed with objective and subjective measures. Objective behavioral tests mainly focus on a subcomponent of IA that has been termed interoceptive sensitivity (Critchley et al., 2004) or interoceptive accuracy (Farb et al., under review), which denotes the objective ability of a subject to accurately perceive inner bodily signals, such as the heartbeat (Brener and Jones, 1974; Whitehead et al., 1977; Schandry, 1981), breathing (Davenport et al., 2007), or gastric activity (Herbert et al., 2012b). Findings up to now suggest that accurate perception of the heartbeat is not increased in meditators (Nielsen and Kaszniak, 2006; Khalsa et al., 2008; Melloni et al., 2013; Parkin et al., 2013) and accurate perception of breathing, was only slightly better in experienced meditators compared to non-meditating controls (Daubenmier et al., 2013). Thus, the notion that contemplative practice profits the interoceptive sensitivity component of IA is put into question by the current state of objective empirical findings.

Besides their obvious problems, subjective measures, such as questionnaires, have the advantage that they more easily allow a broader assessment of IA, covering not only sensitivity to body signals, but also connected beliefs, attitudes, thoughts, and emotions. However, most standardized questionnaires do not fully use this potential, either because they assess only one dimension of IA (e.g., Body Awareness Questionnaire, Shields et al., 1989; Private Body Consciousness Scale, Miller et al., 1981) or because they assess different aspects together in one scale (see Mehling et al., 2009, for a review). Thus, these questionnaires are not likely to accurately depict change in IA elicited by contemplative practice, which has been described as a multidimensional process (Mehling et al., 2011). To overcome this problem, Mehling et al. (2009) constructed a self-report instrument for IA, based on an extensive literature review of published body awareness questionnaires, focus groups with experienced practitioners of mind-body-practices, and extensive psychometric testing (Mehling et al., 2012). The result of these investigations is the multidimensional assessment of interoceptive awareness (MAIA, Mehling et al., 2012), a 32-item self-report measure that measures IA on eight dimensions. One dimension, “Noticing,” reflects the self-reported propensity to become aware of one’s body sensations, such as heartbeat and breath. The other seven dimensions include regulatory aspects of body awareness, that is, how the body and its felt sensations are internally ‘used’ by the subject (to regulate attention or distress, or to gain insight about emotions); reactive aspects, that is, how people respond to body sensations (e.g., with worry or distraction); the awareness of the connection between body sensations and emotional states, and the extent to which the body is experienced as a comforting place, as safe and trustworthy (see **Table 1** for a full description of the dimensions).

**Table 1:** Scales and sample items of the multidimensional assessment of interoceptive awareness (MAIA).

Scale name	Description	Sample questions
Noticing	Awareness of uncomfortable, comfortable, and neutral body sensations	*I notice changes in my breathing, such as whether it slows down or speeds up.*
Not-Distracting	Tendency not to ignore or distract oneself from sensations of pain or discomfort	*I distract myself from sensations of discomfort.*
Not-Worrying	Tendency not to worry or experience emotional distress with sensations of pain or discomfort	*I start to worry that something is wrong if I feel any discomfort.*
Attention Regulation	Ability to sustain and control attention to body sensations	*I can refocus my attention from thinking to sensing my body.*
Emotional Awareness	Awareness of the connection between body sensations and emotional states	*I notice how my body changes when I am angry.*
Self-Regulation	Ability to regulate distress by attention to body sensations	*When I feel overwhelmed I can find a calm place inside.*
Body Listening	Active listening to the body for insight	*I listen for information from my body about my emotional state.*
Trusting	Experience of one’s body as safe and trustworthy	*I feel my body is a safe place.*


The link between contemplative training and the MAIA has been explored in a recent study in which patients with lower back pain were categorized into subjects with and without meditation experience (mixed styles). Higher scores on four of the eight MAIA scales were shown for patients with meditation experience of any kind compared to meditation naïve controls, with strongest effects in Self-Regulation (Mehling et al., 2014). These findings are, however, limited by the cross-sectional nature of the study, the high heterogeneity of meditation practices, and the specific population (pain patients). Sze et al. (2010) studied IA cross-sectionally in Vipassana meditators compared to controls, using three IA scales that mostly measure Noticing, and found higher scores in meditators. Similarly, Noticing aspects of IA have been shown to be increased in yoga practitioners (Rani and Rao, 1994; Daubenmier, 2005; Impett et al., 2006). Three prospective, qualitative studies (Landsman-Dijkstra et al., 2004; Morone et al., 2008; Schure et al., 2008), using content analyses of journal entries and open questions, have investigated changes in IA elicited by mindfulness based stress reduction (MBSR; with slight modifications), and a Body Awareness Program (including mindfulness meditation). Overall, participants reported increased awareness of their body sensations, while stressing many corollary benefits of the practice, such as improved attention, increased awareness of emotions and mind-body-interactions, and a higher propensity to listen to their bodies for insight about their emotional state, particularly when in distress. To summarize, there is cross-sectional evidence for differences in the Noticing aspect of IA in meditators, and qualitative evidence from mostly short-term longitudinal studies about training-induced changes in many other aspects of IA. Up to the present point, there is, however, to our knowledge, no published study on training-related changes in IA based on (a) a well-controlled longitudinal design, (b) a focused mental training program targeting specifically the cultivation of IA, and (c) the assessment of IA through a standardized self-report instrument allowing for the differential measurement of change on different aspects of IA.

To close this gap, we used the MAIA to investigate how mental training influences different dimensions of IA. We investigated this in the context of the “Presence” module of the ReSource Project, a large-scale longitudinal mental training study, conducted by the Max Planck Institute for Human Cognitive and Brain Sciences in Berlin and Leipzig. The ReSource project appeared to be particularly appropriate for such an investigation, because it relies on a large sample (*n* = 148) that, in the first 3-months training module, underwent an intervention, which was specifically designed to cultivate IA through daily practices of a “Body Scan” (BoS) and a “Breath Meditation” (BrM; see Materials and Methods). Both practices are designed to strengthen participants’ focus on body sensations as a vehicle to return to the present moment whenever the mind has wandered. Comparison of the training group with a retest control group, that is, a group that undergoes the same testing but without intervention, allows us to investigate whether IA is altered through mental training and not through familiarity with the scale alone, and if so, which aspects of IA are particularly affected by contemplative, interoceptively focused training.

## MATERIALS AND METHODS

### ETHICS

All reported measurements and the ReSource Presence training were part of the ReSource Project, which was approved by the Research Ethics Committee of the University of Leipzig with the number 376/12-ff, and the Research Ethics Committee of the Humboldt University in Berlin (Mathematisch-Naturwissenschaftliche Fakultät II), with the numbers 2013-02, 2013-29, and 2014-10. All participants gave written informed consent prior to their participation.

### SAMPLES

#### Samples for questionnaire validation

A total of 1,076 subjects (345 male; mean age = 38.7, SD = 9.3; age range = 18–59) filled out the MAIA. Participants were recruited from different German cities (Berlin, Leipzig, Ulm), and through an online server of the University of Mannheim (see **Table 2** for sample details). All participants filled out computerized versions of the MAIA, except for the sample from Ulm, who filled it out on paper.

**Table 2:** Samples for MAIA validation and the intervention study.

No	Description	*n*	Male	Age (SD)	Age range
1	Subjects applying for the ReSource project but not participating in pilot or intervention study (Berlin and Leipzig)	494	176	42.67 (9.7)	20–55
2	Participants of ReSource pilot studies (prior to intervention study, Leipzig)	69	25	33.0 (11.0)	19–55
3	Psychology students (Ulm)	133	3	24.8 (7.4)	18–58
4	Online survey (hosted in Mannheim ^a^)	112	53	32.4 (8.9)	20–59
5	ReSource intervention study participants (training group) (Berlin and Leipzig)	152	73	41.6 (9.4)	20–55
6	ReSource intervention study participants (retest control group) (Leipzig)	80	32	42.3 (8.6)	23–55

	Full sample	1,076	345	38.7 (9.3)	18–59

*The full sample filled out the MAIA and took part in questionnaire validation. Samples 5 and 6 took part in the intervention study as training and control group, respectively. ^a^http://www.forschung-erleben.uni-mannheim.de*

#### Samples in the intervention study

A subsample (*n* = 232; samples 5 and 6 in **Table 2**) took part in the intervention study. 152 subjects (73 male; mean age = 41.6, SD = 9.4; age range = 20–55) were part of the training group, 80 were part of a retest control group (see below). The training group was recruited in a multistep process. Briefly, a total of 2,595 individuals applied for the study, responding to advertisement in newspaper and public transport, to flyers, circulations on relevant e-mail lists, or word of mouth. Participants received extensive information on the study through information evenings and personal phone contact. They were informed that the study would involve daily practice of different mental training exercises, grouped in three modules which aim at training Presence (involving attention and interoception training; the module under investigation in this paper), as well as socio-cognitive and socio-affective abilities. Interested participants were screened via questionnaires and psychological interviews. All participants in the final sample fulfill a number of inclusion criteria, including good psychological and physical health (see Supplementary Material for details).

Eighty subjects (32 male; mean age = 43.3, SD = 8.6; age range = 23–55) served as a retest control group, to account for effects of repeated testing. The sample was recruited from the participant data base of the Max Planck Institute for Human Cognitive and Brain Sciences, Leipzig. Participants in the retest control group did not differ statistically from those in the training group (all *p*s < 0.05) in terms of age, sex, socio-economic status (assessed as income and education level), or any of the MAIA scale values at baseline. For the retest control sample, the study was advertised as an online survey on personality and emotion.

### CONSTRUCTION AND VALIDATION OF THE GERMAN MAIA

#### Translation

As the study took place in Germany and no German version of the MAIA was available, we first translated it. Two of the authors (Wolf E. Mehling and Boris Bornemann, both native German speakers and proficient in English), and a translation agency (Baker and Harrison, Munich, Germany) independently produced German translations of the questionnaire. Wolf E. Mehling and Boris Bornemann then compared the three translations, item by item, and, in the case of different translations, picked the wording that was most easily understandable and closest to the English version. The final questionnaire was then sent to the agency and translated back into English by another independent translator. The back-translation and the original English questionnaire were compared. All items were found to be identical or very similar in wording and meaning so that no further corrections had to be applied.

#### Assessment of psychometric properties

The full sample was used to derive at means, standard deviations, and Cronbach’s alphas for the MAIA scales. Retest reliability was assessed in the retest control group (sample 6, see **Table 2**). For investigation of convergent and discriminant validity, a partial sample (*n* = 268; 122 male, mean age = 41.8, SD = 9.1; age range = 20–55; from samples 1,5, and 6; see **Table 2**) filled out the Five Factor Mindfulness Inventory (FFMQ; Baer et al., 2006; Otto, 2012), which had previously been reported to be positively correlated with the MAIA (Mehling et al., 2012), and a measure of state anxiety (STAI-T from the State-Trait-Anxiety Questionnaire; Spielberger et al., 1970), which had previously been found to be negatively correlated to the MAIA scales (Mehling et al., 2012). We also assessed the Private Body Consciousness Scale (Miller et al., 1981) in 185 subjects (48 male; mean age = 41.8, SD = 9.4; age range = 20–55; from samples 1 and 5; see **Table 2**), to obtain another measure of body awareness. Note that this scale measures exclusively the Noticing facet of IA.

### TRAINING STUDY

#### Training group

The 3-months contemplative intervention was embedded in a large-scale multi-method longitudinal study, the ReSource project. In short, this study consists of several 3-months modules (Presence, Affect, and Perspective). All participants start with a 3-months Presence module aiming at cultivating attention and IA. The 3-months Presence intervention begins with a 3 days silent retreat, in which participants are familiarized with the purpose of the Presence training, and with the two core practices: BoS and BrM. After this introductory retreat, subjects practice alone at home and attend weekly 2-h classes for 13 weeks. Both retreat and weekly classes are facilitated by experienced meditation teachers (nine different teachers with backgrounds in Theravada Buddhism, Tibetan Buddhism, and secularized mindfulness approaches, and long-standing teaching experience). In the weekly classes, the teachers support the participants by supplying additional exercises beside the two core practices (e.g., walking meditation, sound meditation), as well as inspirations and ideas for informal practice in daily life, all aimed at helping the participants to focus their attention and become more aware of their present-moment experience. In addition to the weekly classes, participants are asked to practice five times per week for 30 min (20 min BoS, 10 min BrM) alone at home. These individual home practices are supported by an online platform and a smart phone app, both of which contain guided meditation audio files, recorded by the teachers. Participants were asked to always use the platform or smart phone when meditating, which allowed us to track how often and long they practiced. The teachers followed a secular training protocol developed specifically for the study. Adherence to the protocol was examined by a co-developer of the protocol who attended the daily sessions and by several co-developers who attended the retreat.

The daily core exercise, the BoS and the BrM, have been chosen as they both train attention as well as IA. During the BoS (e.g., Kabat-Zinn, 1990), participants systematically guide their attention to different parts of their body, starting with their toes and ending up on the top of their heads. Participants are asked to attend to the sensations in the various body parts they are focusing on. In the BrM (e.g., Wallace, 2006), participants are asked to focus on the sensations of their breathing. In both practices, participants are asked to resume their interoceptive focus on body parts or their breath, whenever attention has strayed.

Participants of the training group filled out the MAIA twice, once before the retreat (T0) and once after the end of the Presence training (T1; average temporal distance of 113.6 days, SD = 10.7), as part of a larger set of questionnaires. Four participants did not complete the training, reducing the final sample of the training group to 148 subjects. Participants were compensated for their testing times in the ReSource project, granting 7 Euros per hour or part thereof for work on questionnaires. Average time to complete the MAIA was 6:27 (SD = 4:04) minutes at T0 and 5:36 (SD = 4:47) minutes at T1.

#### Retest control group

Participants in the retest group answered the MAIA twice, in an average temporal distance of 113.0 days, SD = 4.3 [not statistically different from the temporal distance in the intervention group, *t*(226) = 0.45, *p* = 0.66], together with other questionnaires, using LIMESURVEY (https://www.limesurvey.org). Participants received 7 Euros per hour or part thereof as compensation for their efforts. Average time to complete the MAIA could not be computed for the retest group due to technical limitations of the survey platform.

### POST-TRAINING QUESTIONNAIRE

After the end of the Presence training, participants filled out a questionnaire containing questions about their appreciation and use of the practice in daily life (BrM and BoS). Here, we use some of these questions as markers of training success. We considered the following questions: “How much did you like [BrM/BoS]?” (1 not at all … 5 a lot), “How difficult was it for you to integrate what you have learned into your everyday life [in week 1–4; 5–8; 9–13]?” (1 very difficult … 5 not difficult at all), “I plan to continue doing [BrM/BoS].” (1 yes | 0 no), “I have looked forward to my daily practice of [BrM/BoS].” “I use what I have learned in everyday life.”, “I think that the time I spend mediating is worthwhile.” (–2 don’t agree at all … +2 fully agree).

## RESULTS

We will first present results on the psychometric properties of the German MAIA. Then, we will report how the MAIA scales are affected by the Presence training. Finally, we will investigate whether the magnitude of change can be predicted by the individual differences in practice time or appreciation of the practices.

### PSYCHOMETRIC PROPERTIES AND VALIDATION

We first analyzed whether the factor structure of the English MAIA would replicate in the German item set. We conducted an exploratory factor analysis (EFA; extraction criterion: eigenvalue > 1; varimax rotation) on the full dataset (*n* = 1,076). The EFA yielded eight factors. These factors group the items in exactly the same manner as in the English version, with the exception of item 19 (“When something is wrong in my life, I can feel it in my body.”), which loaded equally strong on its original factor Emotional Awareness as on Body Listening. We additionally performed a confirmatory factor analysis, which showed that the English factor structure had an acceptable fit to data obtained with the German version, RMSEA = 0.059, CFI = 0.901.

**Table 3** shows mean, standard deviation, and internal consistencies of the German MAIA, across all samples, as well as interscale correlations. All Cronbach’s alphas ranged between 0.56 and 0.89. Alphas of the English MAIA as reported by Mehling et al. (2012) were compared to the present alphas of the German MAIA using the Feldt-Test (Feldt, 1969). Alphas were higher than in the English Version for four scales (Noticing, Attention Regulation, Emotional Awareness, Trusting, *p* < 0.05), lower for one scale (Not-Distracting, *p* < *0*.05), and not statistically different for the remaining three scales (*p* > 0.05).

**Table 3:** Descriptives and internal consistencies of the German MAIA scales.

	*n* = 1,076				*n* = 80	Interscale correlations (*n* = 1,076)						
	Mean	(±SD)	Cronbach’s Alpha	No of items	Retest reliability	Not-Distracting	Not-Worrying	Attention Regulation	Emotional Awareness	Self Regulation	Body Listening	Trust
Noticing	3.36	(0.95)	0.76	4	0.73**	0.19^∗∗^	–0.02	0.43^**^	0.58^**^	0.38^**^	0.45^**^	0.21^**^
Not-Distracting	2.31	(0.89)	0.56	3	0.66**		0.08^*^	0.16^**^	0.13^**^	0.17^**^	0.18^**^	0.15^**^
Not-Worrying	2.61	(1.02)	0.65	3	0.76**			0.24^**^	–0.05	0.20^**^	0.01	0.22^**^
Attention Regulation	2.84	(0.89)	0.89	7	0.72**				0.38^**^	0.62^**^	0.48^**^	0.42^**^
Emotional Awareness	3.32	(1.01)	0.86	5	0.77**					0.46^**^	0.56^**^	0.21^**^
Self Regulation	2.45	(1.07)	0.84	4	0.78**						0.55^**^	0.43^**^
Body Listening	1.99	(1.13)	0.84	3	0.78**							0.33^**^
Trusting	3.43	(1.12)	0.86	3	0.79**							

All scales range from 0 to 5. Retest reliability is computed as Pearson correlation between T0 and T1 scores in the retest control group (sample 6, *n* = 80, see **Table 2**). Interscale correlations are computed as Pearson correlations in the entire sample (*n* = 1,076). *p < 0.05, **p < 0.001.

We investigated convergent and discriminant validity by computing correlations between the MAIA scales and the FFMQ, PBCS, and STAI-Trait (**Table 4**). All MAIA scales show positive or non-significant correlations with the FFMQ scales. Each MAIA scale shows its highest correlation with the FFMQ scale that had shown the highest correlation with that MAIA scale in the English version (see Mehling et al., 2012), except for Not-Distracting (English: AWA, German: DSC). All MAIA scales show positive correlations to the PBSC, except for Not-Worrying, where the correlation is non-significant. All MAIA scales show negative or non-significant correlations with the STAI-T.

**Table 4:** Correlations between MAIA and validation measures.

	FFMQ					PBCS	STAI-T
	OBS	DSC	AWA	NOJ	NOR		
Noticing	0.51***	0.14*	0.02	–0.05	0.13*	0.42***	0.03
Not-Distracting	0.15*	0.22***	0.19**	0.19**	–0.06*	0.17*	–0.11
Not-Worrying	0.11	0.26***	0.25***	0.29***	0.39***	–0.05	–0.43***
Attention Regulation	0.48***	0.23***	0.13*	0.04	0.42***	0.22**	–0.18**
Emotional Awareness	0.56***	0.18*	0.02	–0.08	0.12	0.43***	0.06
Self-Regulation	0.38***	0.19**	0.07	0.06	0.41***	0.26***	–0.24**
Body Listening	0.55***	0.22***	0.04	–0.04	0.23***	0.37***	–0.05
Trusting	0.39***	0.38***	0.24***	0.24***	0.43***	0.20**	–0.44***

*p < 0.05, **p < 0.01, ***p < 0.001.

*FFMQ, Five Factor Mindfulness Questionnaire (OBS-Observing, DSC-Describing, AWA-Acting with Awareness, NOJ-Non-judging, NOR-Non-Reactivity), *n* = 268; PBCS, Private Body Consciousness Scale, *n* = 185 (*n* is smaller, as the PBCS was not assessed in sample 6); STAI-T, Trait Anxiety Inventory, *n* = 268; Descriptively highest correlation in each row is bold.*

### LONGITUDINAL TRAINING-RELATED CHANGES IN MAIA

Adherence to practice was generally high. Participants attended, on average, 11.6 (SD = 1.1) out of the 13 group sessions. Missing of sessions was mostly due to vacations, which subjects were allowed to take while participating in the year-long ReSource project. Outside of the weekly group sessions, participants practiced the BoS (for at least for 20 min) 4.6 times a week (SD = 1.09) and the BrM (for at least 10 min) 4.33 times as week (SD = 1.04), which is only marginally less than they were asked to do (five times a week for each practice). Average total time of meditation practice over the entire Presence training was 36.48 h (SD = 10.85).

**FIGURE 1:**
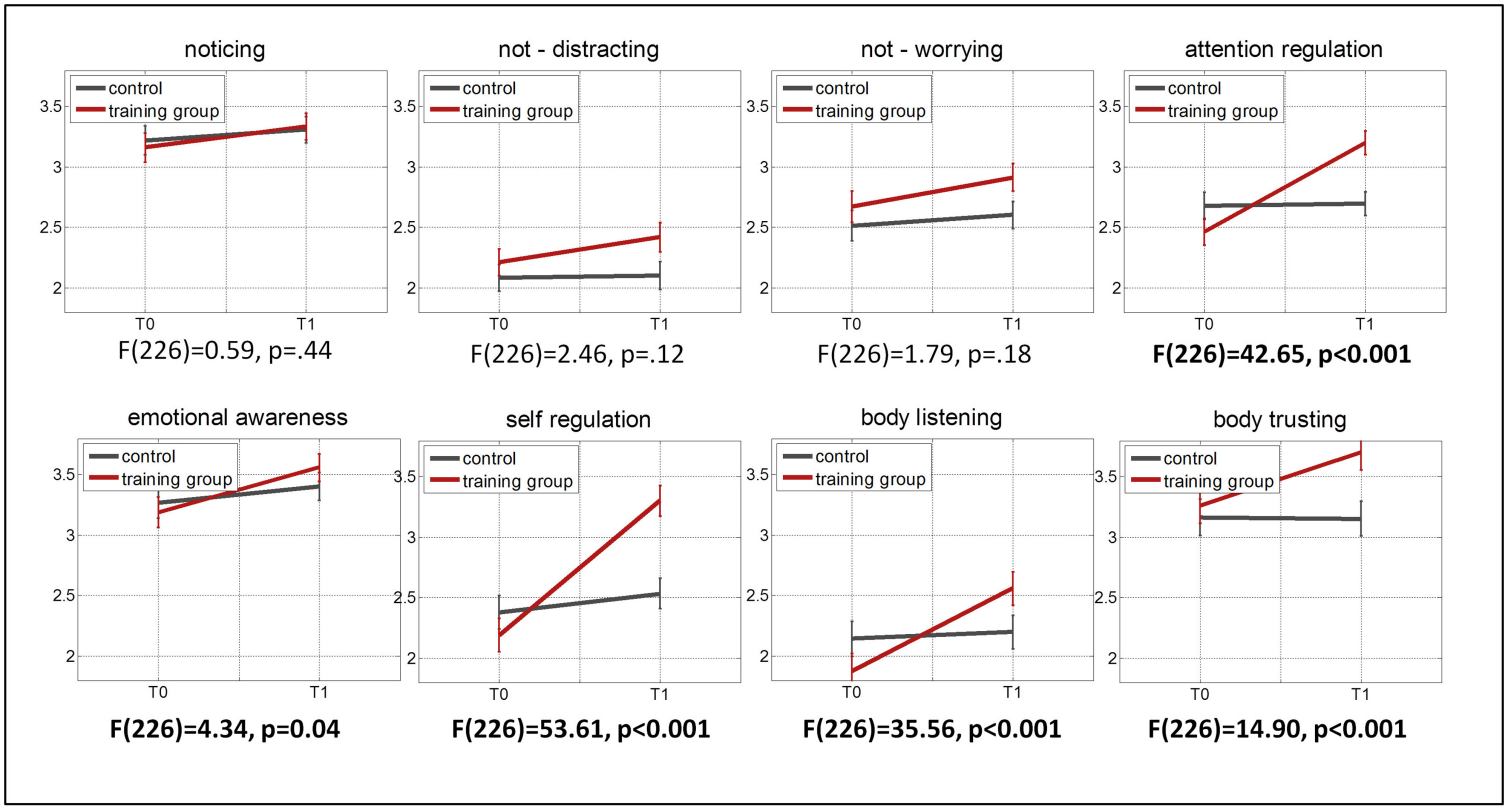
**Changes through Presence Intervention on the eight MAIA scales.** Note: *F*-values are for group*time interactions.

**FIGURE 2:**
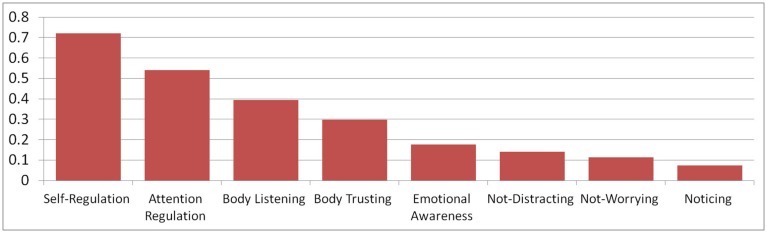
**Effect sizes for changes in the intervention group relative to the control group.** Note: Effect sizes are computed as d = (M_Train_T1_-M_Train_T0_)/[(1/2)(SD_Train_T0_+SD_Train_T1_)] – (M_Control_T1_-M_Control_T0_)/[(1/2)(SD_Control_T0_+SD_Control_T1_)], that is, the mean differences in the training group, standardized by their standard deviation, minus the same measure in the control group (cf. Cohen, 1988).

In the training group, MAIA scores for all scales were significantly higher at follow-up than at baseline (T0), when comparing T0 to T1 values with intra-individual *t-*tests, all *p*s ≤ 0.013 (see **Figure 1**). In the control group, scores did not change significantly (all *p*s ≥ 0.11). The interaction of group and time, tested in a repeated-measures ANOVA, was significant for five out of eight scales, all *F*s ≥ 4.34, all *ps* ≤ 0.04. It was not significant for Noticing, Not-Worrying, and Not-Distracting. **Figure 2** shows the effect sizes of training-related changes, which were computed as mean differences divided by the pooled standard deviation minus the same measure in the control group (Cohen, 1988). The largest effect sizes were found for Self-Regulation (*d* = 0.72), Attention-Regulation (*d* = 0.54), and Body Listening (*d* = 0.40). We also computed the effect size for the PBCS as an alternative body awareness measure. It was *d* = 0.29, expressing a statistically significant change in a within-group *t*-test, *t*(147) = 4.19, *p* < 0.001. The control group did not complete the PBCS; therefore, interaction effects could not be tested. The effect size for Noticing, which measures a similar construct as the PBCS, was in a similar range when not subtracting the control group changes (0.19). For all scales, there was a significant, negative correlation of scale value at T0 with change on that scale (Y1-Y0), with coefficients ranging from –0.18, *p* = 0.016 (Body Listening) to –0.44, *p* < 0.001 (Noticing), indicating that participants with lower initial values showed greater improvements.

There were no significant interactions of time and sex on scale growth (all *p*s ≥ 0.11), showing that both male and female show similar increases on the scales. To confirm that effect sizes did not differ between men and women, *t*-tests of the scale growth (Y1-Y0) comparing men and women were performed, yielding no significant differences (all *p*s ≥ 0.11). When computing the scale growths as baseline corrected T1 values (Residual of Y1 regressed on Y0; see Cohen et al., 2003, p. 375), and again subjecting those values to *t*-tests, women showed stronger improvements on emotional awareness than men, *t*(146) = 1.99, *p* = 0.048. There were no effects of age on scale growth, all *p*s > 0.09.

### DEPENDENCY OF TRAINING EFFECT ON PRACTICE DURATION AND PRACTICE APPRECIATION

Total practice time, that is, how long participants spent meditating throughout the training (assessed by the web platform and smart phone app) predicted increases in Self-Regulation, *r* = 0.18, *p* = 0.027, and Trusting *r* = 0.19, *p* = 0.019, but not on the other scales. When again using a baseline corrected T1 value, predictions became slightly better, with total number of meditations predicting increases in Attention Regulation, *r* = 0.18, *p* = 0.028, Self-Regulation, *r* = 0.22, *p* = 0.007, and Trusting, *r* = 0.20, *p* = 0.013 (see **Table 5**).

**Table 5:** Relations (Pearson correlations) of practice intensity and appreciation to change on the MAIA scales.

	Noticing	Not-Distracting	Not-Worrying	Attention Regulation	Emotional Awareness	Self-Regulation	Body Listening	Trusting
Total practice time (*n* = 147)	0.08	0.05	0.09	0.18**	0.12	0.22**	0.15	0.20*
LikingPractice (*n* = 142)	0.19*	0.04	0.13	0.39***	0.31***	0.43***	0.30***	0.39***
PracticeUse (*n* = 140)	0.34***	0.29***	0.09	0.40***	0.23**	0.32***	0.36***	0.35***
MeditationWorthwhile (*n* = 144)	0.20*	0.08	0.19*	0.23**	0.20*	0.36***	0.15	0.21*

*p < 0.05, **p < 0.01, ***p < 0.001.

*Practice time, that is, total time spent in meditation was automatically assessed using the online platform (webbased + smartphone app). LikingPractice contains both enjoyment of the practice and looking forward to it (see Methods section for exact questions); PracticeUse contains assessment of difficulty to integrate practice in everyday life and a usefulness-of-practice rating. Changes are computed as Y1 residualized at Y0, to account for the baseline-dependency of changes. MAIA change were present for 148 subjects. For each of the variables in the correlation analyses, a few subjects were lost due to technical problems.*

We further investigated whether appreciation of the practices, based on the post-training questionnaire, predicts changes on the MAIA scales. We first examined the structure of participants’ answers in the questionnaire to arrive at aggregated predictors used for subsequent analyses. 97.3% (82.3%) of the subjects stated that they plan to continue practicing BrM (BoS). Because of the lack of variance in the answer to this question it was omitted from further analyses. Scores for the degree of integration of the practices into everyday life in weeks 1–4, 5–8, and 9–13 were intercorrelated (0.56, 0.55, and 0.18, all *p*s < 0.032), and were aggregated into a sum score. This sum score was strongly correlated to the evaluation of the practice as useful in everyday life (*r* = 0.50, *p* < 0.001), so that the two scores could be added into a single variable, called PracticeUse. Looking forward to practice and liking the practice were also highly related for both BoS (*r* = 0.78, *p* < 0.001) and BrM (*r* = 0.56, *p* < 0.001), so we pooled them into variables called “LikingBoS” and “LikingBrM.” Those two scores were uncorrelated, *r* = –0.08, *p* = 0.32, indicating that liking of BrM is independent of liking of BoS. To obtain a global measure of liking of the practices, we added both values into a single score (LikingPractice). LikingPractice and PracticeUse were both mildly to moderately correlated to the evaluation of meditation as a worthwhile activity (0.36 and 0.31, respectively, *p*s < 0.001), so we decided against aggregation of this item into one of the two constructs. We then analyzed how practice appreciation predicts changes in IA on the MAIA. The results can be found in **Table 5**. All correlations became markedly stronger when MAIA scale growths were statistically controlled for baseline levels as described above. We will thus only report correlations with the corrected values. Both LikingPractice and PracticeUse were predictive of changes on the five MAIA scales that showed the strongest intervention effects and on the Noticing scale (*r*s between 0.19 and 0.43, all *p*s < 0.05). PracticeUse additionally predicted changes in Not-Distracting. The evaluation of meditation as a worthwhile activity (PracticeWorthwhile) also predicted changes on six of the eight scales, with slightly lower correlation coefficients. PracticeUse and LikingPractice were intercorrelated, *r* = 0.34, *p* < 0.001, and both were related to total practice time (*r* = 0.23, *p* = 0.005 and *r* = 0.32, *p* < 0.001, respectively).

Regression analyses show that the three appreciation variables (LikingPractice, PracticeUse, PracticeWorthwhile) in combination explained significantly more variance than single predictors alone in several scale changes. For instance, 26% of the variance in changes in Self-Regulation could be explained by a combination of PracticeUse, LikingPractice, and PracticeWorthwhile, *F*(3,135) = 15.80, *p* < 0.001, *p*s ≤ 0.047 for all predictors. Interestingly, total practice time, assessed through the daily practice computer platform, is never a significant predictor of change on any of the MAIA scales when entered into the regression together with the more evaluative questions assessed in the post-training questionnaire.

## DISCUSSION

In the present study, we investigated whether contemplative practice can elicit changes in different aspects of self-reported IA. To this end, we investigated IA in 148 individuals that underwent an intensive 3-months, bodily focused contemplative intervention using a recently developed self-report instrument, the MAIA (Mehling et al., 2012). More specifically, the tested intervention sample was part of a large-scale 1-year longitudinal mental training study, the ReSource project, which began with a 3-months Presence module aiming at cultivating IA and attention through daily practice of two core meditations, a “Breathing Meditation” and a “Body Scan.” As this training project was implemented in Germany, we here also provide a German translation and psychometric validation of the MAIA. In a subsequent step, we investigated whether the ReSource Presence training was able to elicit increases in IA and if so, how different dimensions of IA were differentially affected by the training.

Results show that the German translation of the MAIA has good reliability as well as convergent and discriminant validity. Both are comparable to the English version. Importantly, we give evidence for plasticity in self-reported IA after an intense 3-months bodily focused contemplative training using this new German version of the MAIA. In addition, we could show differential change on the different scales of this self-report measurement. Self-reported Noticing of bodily signals, which is the aspect that previous studies have predominantly investigated using questionnaires and objective measures of interoceptive accuracy, does not show significant changes, whereas other aspects of IA, particularly aspects related to self-regulation, show large training-related changes. Finally, whereas the mere amount of weekly practice sessions predicted training-related changes on the MAIA scales only to a limited extent, more evaluative subjective reports about liking and utilization of the practice in everyday life were stronger predictors of individual differences in training-related changes.

The German version of the MAIA was tested in a sample of 1,076 people within a broad age range. Five of the eight scales showed alphas above 0.8, which is generally regarded as good internal consistency (e.g., George and Mallery, 2003). One scale has acceptable consistency (Noticing, 0.76). Consistency of the remaining two scales is questionable (Not-Distracting, 0.56, and Not-Worrying, 0.65). This suggests that the items of these short three-item scales are heterogeneous. Not-Distracting has a significantly lower internal consistency than in the English version, which points to a potentially problematic translation of the items. Consistencies of Not-Distracting and Not-Worrying are not substantially better in the English version, though, suggesting that the underlying constructs may need more thorough definition and the items need to be adjusted accordingly. With only three items, these scales are also exceptionally short and their consistencies might profit from additional items. Four scales showed significantly higher reliabilities than in the English version. Note, however, that in large samples, also small differences become significant. Only the differences in the consistencies of the Noticing-and the Trusting scale appear numerically meaningful, suggesting that these German scales actually exhibit higher consistencies than those of the English version.

All MAIA scales are related to aspects of mindfulness, as measured by the FFMQ. This was expected, as mindfulness entails the awareness of inner states and processes, of which body sensations are an important part (Baer et al., 2006; Shapiro et al., 2006). The finding lends convergent validity to the MAIA and replicates the results obtained with the English version (Mehling et al., 2012). Also replicating those results, we find all MAIA scales to be either negatively correlated or uncorrelated to trait anxiety, as measured by the STAI-T.

After having established good reliability and validity of the new German MAIA, we could ask our main question whether and how bodily focused contemplative practice would influence different aspects of IA. We could show that participants undergoing a 3-months bodily focused contemplative intervention (the Presence module of the ReSource project) showed increases on five out of eight aspects of IA, when tested in an interaction model including a retest control group that does not undergo any training. These results generally confirm that the MAIA is sensitive to changes in IA through contemplative training. More importantly, however, the multidimensionality of the MAIA also allowed us to test which aspects of IA are particularly affected by the training. Participants show no changes on the Noticing scale (only significant in an intra-individual *t*-test, without comparison to the control group). Noticing is the subjective evaluation of the ability to accurately perceive bodily events. Earlier studies investigating practice-related changes of IA have almost exclusively focused on this aspect. In line with our findings here, effect sizes for changes on this aspect observed in previous studies have been modest. This holds for studies using subjective measures (e.g., Impett et al., 2006) as well as studies using objective measures such as breathing sensitivity (e.g., Daubenmier et al., 2013). We do, however, find significant moderate to large changes (Cohen’s d = 0.40 to 0.72) for the IA sub-components of “Self-Regulation,” “Attention Regulation,” and “Body Listening.” These could be collectively described as the regulatory aspects of IA. They describe how much subjects deliberately focus on their body in order to regulate emotion, attention, and to gain insight about their emotional-motivational states. This quantitative finding echoes the qualitative reports of participants in mind–body interventions who claim profiting from better attention and emotional clarity (Landsman-Dijkstra et al., 2004; Morone et al., 2008; Schure et al., 2008). The finding is also in correspondence with the training method of the ReSource Presence module. Deliberately paying attention to body sensations and redirecting it there when the mind has wandered are at the heart of both core practices (BoS and BrM). Our findings indicate that these practices strengthen participants’ abilities to direct attention toward their bodies (Attention Regulation) and that they make use of these abilities to regulate distress (Self-Regulation) and to gain insight into their emotional-motivational state (Body Listening).

Participants also report a heightened sense of awareness of the connection between bodily and emotional states (Emotional Awareness). This awareness forms the basis for the deliberate use of the body for insight and decision making that is captured in the Body Listening facet described above. An increase on the Emotional Awareness scales dovetails with findings by Sze et al. (2010) who report a higher congruency between the subjective emotional and the objective physiological state (heart rate) in meditators as compared to non-meditating controls. Participants also develop a higher sense of trust in their own body, experiencing their body as a safe place and their sensations as trustworthy, as indicated by increases on the MAIA Trusting scale. It is possible that their frequent sitting in a safe environment while focusing on their bodies turns body sensations into safety cues by means of conditioning. As an alternative hypothesis, one may assume that focusing on body sensations, at least as long as one is healthy, transmits a quality of peace and tranquility, of ‘basic okay-ness’ (Rinpoche and Swanson, 2012), and puts the organism into a grounded, calm, and present-focused ‘being-mode’ (Kabat-Zinn, 1990). The discovery of these qualities inherent in body focus may be responsible for the changes on the Trusting scale. Finally, it is also possible that the acquired skills in using IA for self-regulation and emotional insight spill over into a more general positive attitude toward the body, into an experience of the body as helpful, safe and trustworthy.

The scale Not-Worrying did not improve significantly, when tested in comparison to the control group (changes are only significant in intra-individual *t*-tests). The Presence module of the ReSource study does not explicitly address the topic of dealing with difficult emotions or thoughts. It only encourages participants to attend to present bodily and sensory experiences. A change in worrisome thoughts about the body could thus only have happened incidentally. Results suggest that this happened only to marginal extents. Similarly, Not-Distracting, that is, the tendency not to distract oneself from unpleasant body sensations, did not show significant improvements in comparison with the control group. This is surprising at first glance because, in both practices, participants are asked to direct attention to all body parts and stay with each for a while. Naturally, participants will encounter unpleasant sensations in this process. However, as described above, the Presence training explicitly does not address working with emotions in any way, as this is part of the later affective training module. Our findings thus show that mere training of bodily focus does not suffice to significantly alter mental habits of participants to avoid unpleasant sensations. The effects of the ReSource Presence training may be distinct here from other trainings, such as MBSR, where interoception and attention training is infused with more emotionally focused practice aspects, such as acceptance (e.g., Kabat-Zinn, 1990). Finally, we can also not rule out that the smaller changes in Not-Worrying and Not-Distracting are due to the low reliability of these two scales.

All changes were independent of sex and age (except for slightly higher improvements in emotional awareness for women). Higher initial scale values, however, predicted lower training-induced increases for all scales (= ceiling effect). These findings indicate that the Presence training of the ReSource study benefited men and women, old and young people alike with regard to IA, but may have particularly benefited participants who started off with lower baseline values. The magnitude of the latter effect could potentially be inflated through regression to the mean (Bland and Altman, 1994).

Growth on the MAIA scales was only marginally predicted by the mere practice hours, as assessed through our meditation platform during daily individual practice. Reliable correlations were only found for growth on Attention Regulation, Self-Regulation, and Trusting, and those were relatively small (∼0.2). This may have to do with the generally strong adherence of all ReSource participants to the required daily practice, resulting in low variance of practice hours. Recent studies and meta-analyses have also found dose dependent effects to be very small or even absent in meditation based interventions (Carmody and Baer, 2009; Hofmann et al., 2010; Jensen et al., 2012). However, in these studies, variance in training dose may not have been big enough to predict individual differences in change. Clearly, several cross-sectional studies in long term meditators found moderately sized correspondence between lifetime practice hours and outcome variables such as interospective abilities (Fox et al., 2012), and brain structure (Lazar et al., 2005), and some longitudinal training studies also demonstrate dose-dependent effects (e.g., Carmody and Baer, 2008; Pace et al., 2009; Rosenzweig et al., 2010). Additionally though, changes through this type of intervention may depend on a good match of person and practice, or the integration of the practice into everyday life. And indeed, the analyses of participants’ ratings, derived via questionnaire after the training, revealed that liking of the practice shows moderately sized correlations with training-related increases on six of the eight scales. This makes sense, as all practice-induced changes tend to be stronger, when the practice is embraced with emotional inclination and verve (Pekrun et al., 2002; Hu et al., 2007). The measure “Use of the practices in daily life” from the post-training questionnaire even predicts individual differences in increases in IA on seven out of the eight scales. This is in line with contemplative advice to put strong emphasis on practicing in everyday life, as most time is spent outside of formal practice and, ultimately, it is everyday life that the transformation is targeted for (Williams and Penman, 2011). Our findings suggest that contemplative training may become more effective, at least in fostering IA, if participants enjoy the training and the exercises and if the practices are tailored in ways that they are easy to integrate into everyday life.

### LIMITATIONS

The current study uses self-report to assess participants’ IA. It is not clear to which extend self-reported IA corresponds to IA as assessed through objective measures. Future research has to follow up on this question, bearing in mind the multi-dimensionality of the construct. Thus, in exploring the question, several objective tests (using, e.g., behavioral, physiological, and neuronal parameters) are needed which can assess the different aspects of IA as measured through the MAIA. Some aspects, such as Not-Worrying, are inherently difficult to assess objectively. Still, more ecologically embedded methods, such as experience sampling, may help arriving at measures which are less susceptible to cognitive biases and the challenges of comparing oneself to other individuals (a challenge that is even bigger for covert traits such as IA). In the original publication of the MAIA, Mehling et al. (2012) acknowledge these problems and follow that “it [the MAIA] is largely capturing intra- rather than interindividual variability.” We can thus conclude from the results that the benefits participants subjectively experience through the devised contemplative training lie in the regulatory aspects of body awareness to much stronger extents than in the mere Noticing aspect. We consider this by itself very informative. Whether and how strongly these changes are objectively induced can, however, not be clarified by the current study.

Participants may have also answered the MAIA according to demand characteristics. After 3 months of bodily focused contemplative training, they may expect that their IA should have changed and answer accordingly. Participants in the training group, who enrolled for an intervention study, may also have had different expectations from those in the control group, who enrolled for a questionnaire study. While these possibilities need to be considered, it is interesting to note that participants do not report changes in Noticing, which is the most obvious skill expected to have increased after 3 months of bringing attention to the body. Instead, they report using IA to regulate distress (Self-Regulation), a strategy which has not been actively encouraged in the training, but which participants seem to incidentally adopt. This speaks against the adherence to obvious expectations but rather suggests that participants report on their actual experience. Furthermore, changes in the answering behavior of participants may have occurred due to an altered understanding of the MAIA items. Such change in the understanding of language describing inner states and processes through meditation practice has been discussed by Grossman (2008) in relation to mindfulness questionnaires. It may also pertain to the MAIA.

Finally, our training sample only comprised psychologically and physiologically healthy individuals. It remains an open question, how a contemplative intervention as that of the current study affects body awareness in participants with mental or physical problems.

## CONCLUSION

We used a multidimensional self-report instrument to study longitudinal changes in IA through a contemplative intervention. We observed training-related changes on five out of eight aspects of self-reported IA, when tested in comparison to a retest control group that underwent the same assessment but did not receive any training. Importantly, the multidimensional assessment reveals a certain profile of changes: changes in the self-reported ability to notice bodily changes, such as changes in breathing or heartbeat, are not statistically significant. This is the facet that has been predominantly investigated by previous studies, many of which have yielded only marginal or null-findings. Moderate to large effects are, however, observed for regulatory aspects of IA, that is, how the body is used for self-regulation and emotional insight. The study thus elucidates in which ways contemplative mind-body-practices are most transformative and which facets of IA are only marginally affected. It underscores the need for multidimensional assessment of IA, particularly when interested in changes through contemplative practice. As a quantitative study on subjective reports, this study opens future research directions along several methodological pathways. First, objective tests could be utilized to assess those dimensions of IA which have shown the strongest change through the training (e.g., down-regulation of objective distress markers through awareness of body sensations). Second, qualitative research (e.g., using elicitation interviews, Petitmengin, 2006), could dig deeper into the ways in which participants’ relationship to their bodies, their sense of embodiment, or their deliberate use of body-focus is altered through the training. Third, future studies should resolve the question how different facets of IA as assessed by self-report correlate and interact with objective measures of interoception, such as interoceptive accuracy.

Given the relevance that interoception has for psychological and physical health, this study has important implications. It shows that mental training that involves focus on body sensations improves several aspects of IA, and particularly that it strengthens participants’ use of body sensations to become more aware of emotions and to regulate distress. Such a training program thus seems advisable as a way to foster emotional clarity and well-being in healthy individuals. It may also be helpful for clinical populations suffering from difficulties in emotion recognition or distress regulation, such as in alexithymia, affective and anxiety-disorders, or patients with aggressive-impulsive behavior.

## Conflict of Interest Statement

The authors declare that the research was conducted in the absence of any commercial or financial relationships that could be construed as a potential conflict of interest.
