# Supplementary material for: Differential changes in self-reported aspects of interoceptive awareness through 3 months of contemplative training
Source: Front Psychol. 2015 Jan 6;5:1504. doi: 10.3389/fpsyg.2014.01504 (PMC4284997; doi:10.3389/fpsyg.2014.01504)
Supplement: Supplementary file 1 [file Presentation_1.PDF]

Multidimensional Assessment of Interoceptive Awareness (MAIA)

Deutsche Fassung

Download und aktuelle Informationen:
http://www.osher.ucsf.edu/maia/

Benutzerrecht und Copyright

Der MAIA ist urheberrechtlich geschützt. Gleichwohl ist seine Benutzung gebührenfrei und bedarf keiner ausdrücklichen schriftlichen Genehmigung. Mit seiner Anwendung erkennt man die folgenden Regeln an:

- Bei Bezugnahme auf den Fragebogen bitten wir den vollen Namen zu nennen: Multidimensional Assessment of Interoceptive Awareness.
- Modifikationen sind ohne unser schriftliches Einverständnis erlaubt. Allerdings bitten wir darum, dass jegliche Veränderung in den entsprechenden Publikationen klargestellt und uns mitgeteilt wird.
- Falls Sie Teile des MAIA benutzen wollen, empfehlen wir nicht Einzelfragen sondern komplette Subskalen zu verwenden, damit die psychometrischen Eigenschaften erhalten bleiben.
- Bitte senden Sie uns eine Kopie, falls Sie eine Übersetzung in eine Fremdsprache vornehmen.
- Falls sich andere Kollegen für den MAIA interessieren, verweisen Sie bitte auf die Originalpublikation (PLoS-ONE 2012), die der deutschen Version (Frontiers 2015) und die Website www.osher.ucsf.edu/maia/, damit sie die jüngste Version und Scoring-Anleitung erhalten.

Scoring-Anleitung

Kalkulieren Sie den Mittelwert (mean) sämtlicher Antwort-Werte einer Subskala.

Bitte beachten Sie: Die Werte für die Fragen 5, 6 und 7 für Nicht-Ablenken, und die Fragen 8 und 9 für Sich-Keine-Sorgen-Machen werden umgekehrt.

1. Bemerken: Gewahrsein von unangenehmen, angenehmen und neutralen Körperempfindungen.

   F1___ +F2___ +F3___ +F4___/4 = _____

2. Nicht-Ablenken: Die Tendenz, unbequeme Empfindungen nicht zu ignorieren oder sich nicht von ihnen abzulenken

   (5 - F5)___ + (5 - F6) ___ + (5 - F7)___ /3 = _____

3. Sich-Keine-Sorgen-Machen: Die Tendenz, sich bei Schmerz oder unbequemen Empfindungen keine Sorgen zu machen und nicht in emotionalen Stress zu geraten

   (5 - F8)___ + (5 - F9)___ +F10 /3 = _____

4. Aufmerksamkeits-Steuerung: Die Fähigkeit, Aufmerksamkeit auf Körperempfindungen zu lenken und zu bewahren

   F11___ + F12___ + F13___+ F14___ + F15___ + F16___ + F17___ /7 = _____

5. Emotionales Gewahrsein: Das Gewahrsein eines Zusammenhanges von Körperempfindungen mit Emotionen

   F18___ + F19___ + F20___ + F21___ + F22___ /5 = _____

6. Selbst-Regulation: Die Fähigkeit, Ungemach zu regulieren, indem die Aufmerksamkeit Körperempfindungen zugewendet wird

   F23___ + F24___ + F25___ + F26___ /4 = _____

7. Auf-den-Leib-Hören: Das aktive Auf-den-eigenen-Leib-Hören um von ihm zu lernen

   F27___ + F28___ + F29___ /3 = _____

8. Vertrauen: Den eigenen Leib als sicher und vertrauenserweckend erfahren

   F30__ + F31___ + F32___ /3 = _____

Unten finden Sie eine Liste von Aussagen. Geben Sie bitte für jede der folgenden Aussagen an, wie oft sie generell im täglichen Leben auf Sie zutrifft.

	**Kreisen Sie in jeder Reihe eine Zahl ein**					
	**Nie**					**Immer**
1. Wenn ich angespannt bin, merke ich wo in meinem Körper die Anspannung auftritt.	0	1	2	3	4	5
2. Ich merke es, wenn ich mich in meinem Körper nicht wohlfühle.	0	1	2	3	4	5
3. Ich merke, wo in meinem Körper ich mich wohlfühle.	0	1	2	3	4	5
4. Ich bemerke Veränderungen in meiner Atmung, zum Beispiel ob ich langsamer oder schneller atme.	0	1	2	3	4	5
5. Ich ignoriere körperliche Anspannung oder Unwohlsein bis diese stärker werden.	0	1	2	3	4	5
6. Ich lenke mich von unangenehmen Empfindungen ab.	0	1	2	3	4	5
7. Wenn ich Schmerz oder Unbehagen empfinde, versuche ich mich durchzubeißen.	0	1	2	3	4	5
8. Wenn ich körperliche Schmerzen habe, ärgere ich mich.	0	1	2	3	4	5
9. Wenn ich mich unwohl fühle, fange ich an mir Sorgen zu machen, dass irgendetwas nicht stimmt.	0	1	2	3	4	5
10. Ich kann unangenehme Körperempfindungen spüren, ohne dass sie mich beunruhigen.	0	1	2	3	4	5
11. Ich kann auf meine Atmung achten ohne von dem, was um mich herum geschieht, abgelenkt zu werden.	0	1	2	3	4	5
12. Ich kann meiner inneren Körperempfindungen gewahr bleiben, auch wenn um mich herum eine Menge los ist.	0	1	2	3	4	5
13. Ich kann auf meine Körperhaltung achten, während ich mich mit jemandem unterhalte.	0	1	2	3	4	5
14. Wenn ich abgelenkt bin, kann ich mit meiner Aufmerksamkeit zu meinem Körper zurückkehren.	0	1	2	3	4	5
15. Ich kann meine Aufmerksamkeit vom Denken auf das Spüren meines Körpers zurücklenken.	0	1	2	3	4	5
16. Ich kann den gesamten Körper auch dann weiter bewusst wahrnehmen, wenn ich in einem Teil Schmerz oder Unbehagen empfinde.	0	1	2	3	4	5

Geben Sie bitte für jede der folgen Aussagen an, wie oft sie generell im täglichen Leben auf Sie zutrifft.

	Kreisen Sie in jeder Reihe eine Zahl ein					
	**Nie**					**Immer**
17. Ich kann meine Aufmerksamkeit bewusst auf meinen Körper als Ganzes richten.	0	1	2	3	4	5
18. Ich bemerke, wie mein Körper sich verändert, wenn ich wütend bin.	0	1	2	3	4	5
19. Wenn etwas in meinem Leben nicht stimmt, kann ich das in meinem Körper spüren.	0	1	2	3	4	5
20. Ich merke, dass mein Körper sich anders anfühlt, wenn ich etwas friedliches und entspannendes erlebe	0	1	2	3	4	5
21. Ich merke, dass meine Atmung freier und leichter wird, wenn ich mich wohlfühle.	0	1	2	3	4	5
22. Ich merke, wie mein Körper sich verändert, wenn ich glücklich oder fröhlich bin.	0	1	2	3	4	5
23. Wenn mir alles zu viel wird, kann ich einen Ort der Ruhe in mir finden.	0	1	2	3	4	5
24. Wenn ich meine Aufmerksamkeit auf meinen Körper richte, empfinde ich ein Gefühl innerer Ruhe.	0	1	2	3	4	5
25. Ich kann meinen Atem dazu benutzen, innere Spannungen abzubauen	0	1	2	3	4	5
26. Wenn ich in meine Gedanken verstrickt bin, kann ich meinen Geist beruhigen, indem ich auf Körper und Atem achte.	0	1	2	3	4	5
27. Ich höre auf meinen Körper, was er über meine emotionale Verfassung sagt.	0	1	2	3	4	5
28. Wenn ich aufgebracht bin, nehme ich mir Zeit herauszufinden, wie mein Körper sich anfühlt.	0	1	2	3	4	5
29. Ich höre auf meinen Körper um zu erkennen was zu tun ist.	0	1	2	3	4	5
30. Ich bin in meinem Körper zu Hause.	0	1	2	3	4	5
31. Ich empfinde meinen Körper als einen sicheren Ort.	0	1	2	3	4	5
32. Ich vertraue meinen Körperempfindungen.	0	1	2	3	4	5
