# Supplementary material for: Differential changes in self-reported aspects of interoceptive awareness through 3 months of contemplative training
Source: Front Psychol. 2015 Jan 6;5:1504. doi: 10.3389/fpsyg.2014.01504 (PMC4284997; doi:10.3389/fpsyg.2014.01504)
Supplement: Supplementary file 2 [file Presentation_2.PDF]

Supplementary Material to

Differential changes in self-reported aspects of interoceptive awareness through three months of contemplative training

Boris Bornemann[1], Beate M. Herbert[2,3], Wolf E. Mehling[4], Tania Singer[1] *

[1] Department of Social Neuroscience, Max Planck Institute for Human Cognitive and Brain Sciences, Leipzig, Germany

[2] Beate M. Herbert, Clinical Psychology and Psychotherapy, University of Tuebingen, Germany

[3] Health Psychology, University of Ulm, Germany

[4] Department of Family and Community Medicine, Osher Center for Integrative Medicine, University of California San Francisco, USA

*** Correspondence:** Tania Singer, Max Planck Institute for Human Cognitive and Brain Sciences, Department of Social Neuroscience, Stephanstrasse 1a, 04103 Leipzig, Germany, singer@cbs.mpg.de, Tel. +49 341 9940 149

A) Exclusion criteria for participants in the ReSource study
B) Exploratory Factor analysis of the MAIA - Rotated Component Matrix
C) References

A) Exclusion criteria for participants in the ReSource study

Participants were not admitted for the study if the fulfilled any of the following criteria, assessed through self-report:

- not between 20 and 55 years old
- no computer access at home / no internet connection
- not meeting our MRI safety house standards (no irremovable metal in the body, tattoos in the upper part of the body, permanent make-up, pregnancy or being in the lactation period, obesity, diabetes, neurological disorders, head trauma with loss of consciousness, peripheral vascular diseases, peripheral arterial diseases, Reynaud's diseases, involuntary motor disorders, epilepsy, insulin pumps, retainer, inner ear implants, pacemakers, drug pumps, cerebral water drainage)
- regular spiritual practice in the last 2 years
- regular meditation practice in the last 2 years, participation in meditation retreats
- does not speak/understand German fluently
- chronic pain
- psychotherapy in the last 2 years
- allergic to adhesive tape
- smoking more than 5 cigarettes a week
- drugs and alcohol abuse
- diagnosed mental disorders (ok if recovered more than two years ago)
- cortisol intake
- medications (that affect central nervous system function, psychotropics, opiates, corticosteroids, medications for anxiety, depression, or other psychological problems)
- studies or studied psychology

Additionally, we screened our participants for mental disorders, using the following Health Questionnaires:

- Major (ICD-10) Depression Inventory (MDI), exclusion from mild to severe;
- Toronto Alexithymia Scale (TAS-20), exclusion if > 60;
- State-Trait-Angstinventar; STAI X2-Trait, exclusion if > 56;
- Patient Health Questionnaire-D (PHQ-D), see manual for analyses;
- Prescreening question for the Structured Clinical Interview for DSM-IV for axis II personality disorders (SCID-II), as a basis for an psychological interview;

Upon fulfillment of all criteria, participants were additionally screened for psychological health in an interview by a trained clinical psychologist. This interview included a computer-assisted version of the Structured Clinical Interview for DSM-IV Axis-I disorders (SCID-I) (First et al., 2012), the DIA-X (Wittchen & Pfister, 1997) and the German version of the SCID-II for Axis-II disorders (Gibbon et al., 1997; Wittchen et al., 1997). Participants were excluded if they currently or in the past two years fulfilled the criteria for an Axis-I disorder, if they at any point fulfilled the criteria for schizophrenia or other psychotic disorders, bipolar disorder or a substance-dependence or if they fulfilled the criteria for an Axis-II disorder.

B) Exploratory Factor analysis of the MAIA - Rotated Component Matrix

	1	2	3	4	5	6	7	8
MAIA001	.091	.128	.174	**.751**	-.009	.262	.010	-.010
MAIA002	.111	.226	-.063	**.716**	.016	.103	.092	-.075
MAIA003	.172	.138	.159	**.768**	.169	.053	-.024	-.070
MAIA004	.308	.273	.099	**.518**	.013	.057	.006	-.047
MAIA005	-.216	-.067	.004	-.224	-.123	-.081	.060	**.620**
MAIA006	-.044	-.060	.033	.015	-.026	-.061	.108	**.746**
MAIA007	.093	.032	-.130	.000	.003	.012	-.088	**.750**
MAIA008	-.045	.007	-.087	-.005	-.040	.010	**.647**	.254
MAIA009	-.085	.057	-.014	.115	-.055	.046	**.842**	.022
MAIA010	.202	-.009	.076	.040	.137	-.022	-**.749**	.212
MAIA011	**.705**	.100	.239	.070	-.011	.125	-.111	-.042
MAIA012	**.731**	.089	.134	.147	.133	.176	-.080	-.062
MAIA013	**.719**	.123	.011	.026	.157	.067	-.021	-.004
MAIA014	**.816**	.079	.144	.102	.138	.167	-.057	-.061
MAIA015	**.754**	.081	.272	.152	.128	.097	-.036	-.036
MAIA016	**.675**	.014	.148	.130	.137	.086	-.104	.015
MAIA017	**.603**	.175	.282	.169	.161	.070	-.065	-.011
MAIA018	.253	**.487**	-.039	.281	.023	.351	-.011	.018
MAIA019	.120	**.489**	-.055	.311	-.002	.493	.110	.036
MAIA020	.097	**.822**	.184	.181	.079	.133	.017	-.052
MAIA021	.148	**.807**	.258	.165	.041	.086	.015	-.061
MAIA022	.092	**.837**	.168	.188	.100	.200	.033	-.028
MAIA023	.232	.266	**.560**	.008	.247	.160	-.040	.029
MAIA024	.238	.207	**.728**	.057	.235	.153	-.085	-.035
MAIA025	.371	.106	**.713**	.172	.059	.139	-.068	-.089
MAIA026	.366	.113	**.720**	.130	.054	.195	-.083	-.052
MAIA027	.207	.183	.167	.178	.093	**.781**	.064	-.105
MAIA028	.240	.183	.378	.124	.093	**.670**	.000	-.117
MAIA029	.197	.191	.203	.104	.169	**.772**	.005	-.001
MAIA030	.203	.039	.132	.034	**.878**	.057	-.056	-.044
MAIA031	.212	.015	.196	.016	**.867**	.067	-.122	-.027
MAIA032	.177	.131	.069	.113	**.746**	.143	-.072	-.078

Extraction Method: Principal Component Analysis.
Rotation Method: Varimax with Kaiser Normalization.

References

First, M. B., Spitzer, R. L., Gibbon, M., & Williams, J. B. (2012). *Structured Clinical Interview for DSM-IV Axis I Disorders (SCID-I), Clinician Version, Administration Booklet*: American Psychiatric Pub.

Gibbon, M., Spitzer, R. L., & First, M. B. (1997). *User's guide for the structured clinical interview for DSM-IV axis II personality disorders: SCID-II*: American Psychiatric Pub.

Wittchen, H. U., & Pfister, H. (1997). *Diagnostisches Expertensystem für psychische Störungen (DIA-X).* . Frankfurt: Swets & Zeitlinger.

Wittchen, H. U., Zaudig, M., & Fydrich, T. (1997). *SKID-Strukturiertes Klinisches Interview für DSM-IV. Achse I und II*. Göttingen: Hogrefe.
